# Recurrent Bronchitis*Read before the Hill County Medical Society Friday, December 10, 1915.

**Published:** 1915-12

**Authors:** L. P. Tenny

**Affiliations:** Troup, Texas


### Recurrent Bronchitis.*

DR. L. P. TENNY, TROUP, TEXAS.

I shall limit this paper to a discussion of that phase of our subject which affects childhood, for it is essentially a disease of childhood, and a typical case of recurrent bronchitis is rarely encountered in an adult.

Also, I wish, at the outset, to draw a distinction between this affection and chronic bronchitis. Recurrent bronchitis is not a chronic affection but a recurrence of acute attacks in which there is a peculiar susceptibility, which can only be accounted for by

*Read before the Hill County Medical Society Friday, December 10, 1915.

the existence of the rheumatic diathesis. Those cases that have come under my observation have been otherwise healthy, active, robust children.

The etiology of this condition is more or less obscure. I am forced to believe that heredity plays an important rôle; at least, the rheumatic tendency is transmitted from parent to child. I recall one case in point in which the father, for a number of years, was a confirmed asthmatic; following a move to a different climate he was relieved and for a number of years has been entirely free from asthma. Two children of this man developed recurrent bronchitis at an early age, and have had numerous attacks. Two other children in the same family and living under the same environments are perfectly free from any tendency to bronchial trouble.

There is no doubt in my mind that the immediate attacks are precipitated by an overindulgence in sweet stuffs. As a rule, those children who are subject to repeated attacks of bronchitis are perfect fiends for sugar. My attention was first called to this by reading Dr. Kerley's valuable book on diseases of children; since then I have had abundant opportunity of confirming this truth. My limited experience does not bear out the further statement of Dr. Kerley that an excessive diet of red meats is also responsible for many of these acute attacks.

The symptoms of recurrent bronchitis are readily recognizable, being identical with those of simple acute bronchitis; only, perhaps, at times more exaggerated. The child, otherwise healthy, will suddenly develop a simple acute coryza, which rapidly extends to the upper bronchial tubes; a harsh brassy cough develops, respiration becomes rapid and shallow; many rales can be heard all over the lung area; the temperature rises to 101° F. or 102° F., rarely higher. The area of involvement, at times, is so extensive that one fears the development of lobular pneumonia; in fact, I have, several times, felt almost sure that I had a typical case of broncho-pneumonia, when suddenly all symptoms subside and the child is, within a very short time, practically well. Usually these attacks will cover a period of from three to five or six days, occasionally lasting as long as ten days.

In a typical case of recurrent bronchitis these attacks are repeated, sometimes at intervals of two to three weeks, and are always more severe in the winter and spring months, when the child is confined to the house more closely, though I have seen them occur in the midst of summer.

Treatment is directed to the relief of the attack, and in the interval to prevention of a return of this distressing condition. Treatment of the acute symptoms differs radically from that of acute bronchitis. It is useless and positively harmful to give those little patients a lot of expectorants and syrups. I have tried them all repeatedly, and, if they have any effect at all, it is to aggravate the symptoms.

Ten grains of bicarbonate of soda, begun early in the attack, and repeated every two hours, will do more to cut it short than anything else in my experience. I also give sodium salicylate in doses of 5 grains to 10 grains, according to age, every three or four hours.

If the dyspnea becomes very marked, and breathing labored, steam inhalations of creosote will often alleviate the symptoms and add to the comfort of the patient. I use about ten drops of creosote in a quart of boiling water.

The most important part of our treatment consists in that during the interval to prevent return of the attack. For this purpose we give a combination of bicarbonate and salicylate of soda according to the following formula:

For child 5 to 10 years of age.

| | | |
|---|---|---|
| ℞ | Sodii salicylate .................... | 2 drachms. |
| | Sodii bicarbonate .................. | 2 drachms. |
| | Elix. simplex ...................... | 4 drachms. |
| | Aqua dist., q. s., ad................. | 4 ounces. |

M. Sig.: A teaspoonful in half a glass of water three times daily, after meals.

This is continued for weeks and even months, if necessary. Restriction of the sugar element in the diet must be insisted upon. I believe it is best to absolutely prohibit any sugar until patient is free from this trouble for six months or a year. Of course, it is necessary to keep the eliminative organs active. This may be accomplished best by a morning dose of magnesium sulphate, once or twice a week.

These patients should be encouraged to eat green vegetables and fruits freely. I also find that cod liver oil given in half to one teaspoonful doses, three times daily, and continued during the whole winter and spring, puts our patients in a condition to resist attack. In fact, I regard it as almost a specific in these bronchial affections.

An outdoor life, as far as practicable, should be insisted upon, with moderate exercise.

